# Supplementary figures and images for: Monogenean anchor morphometry: systematic value, phylogenetic signal, and evolution
Source: PeerJ. 2016 Feb 4;4:e1668. doi: 10.7717/peerj.1668 (PMC4783769; doi:10.7717/peerj.1668)

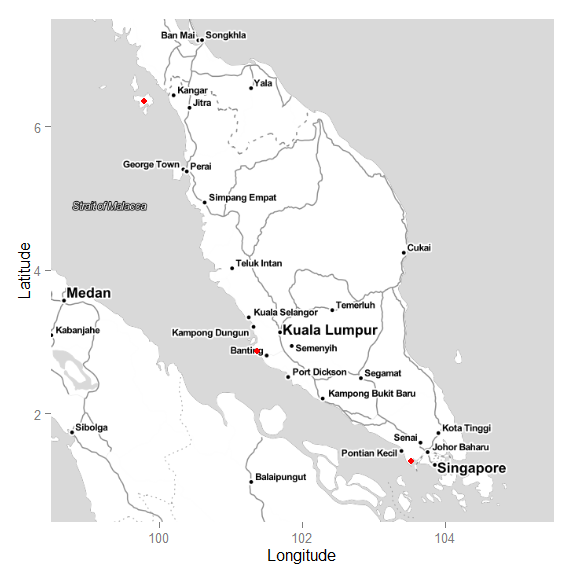

Supplement: Figure S1 — Map of Peninsular Malaysia and the sampling locations (red circles). From north to south: Langkawi Island (6°21′N, 99°48′E; sampling period: 2008–2012), Carey Island (2°52′N, 101°22′E; sampling period: 2009–2012), Sungai Pulai Estuary, Johor (1°20′N, 103°32′E; sampling period: 2013). The map was made using the ggmap R package (Version 2.4; Kahle & Wickham, 2013). Image source: Google Maps. [file peerj-04-1668-s001.png]

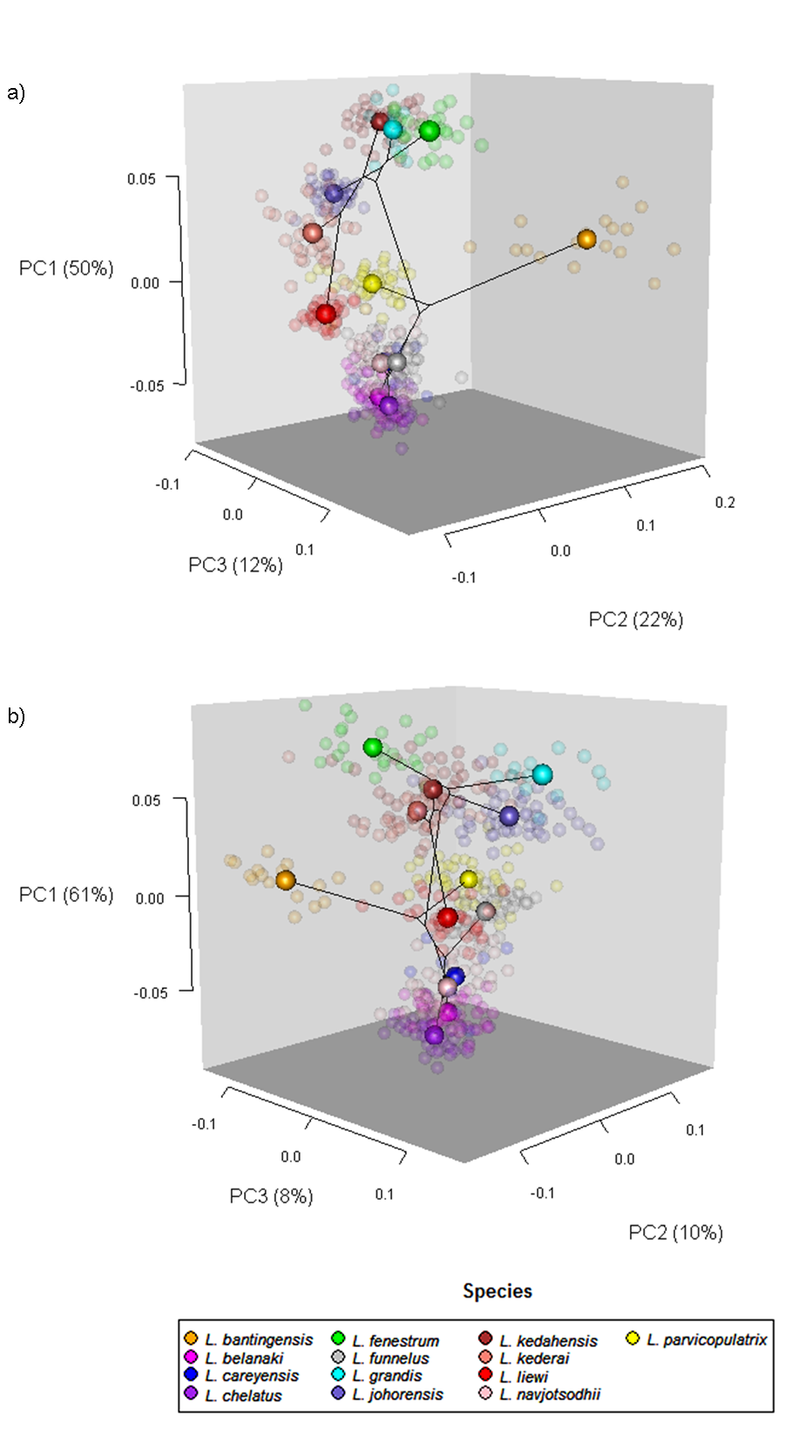

Supplement: Figure S17 — PCA plots of the first three principal components of shape variables for the (A) ventral anchors, and (B) dorsal anchors. [file peerj-04-1668-s017.png]

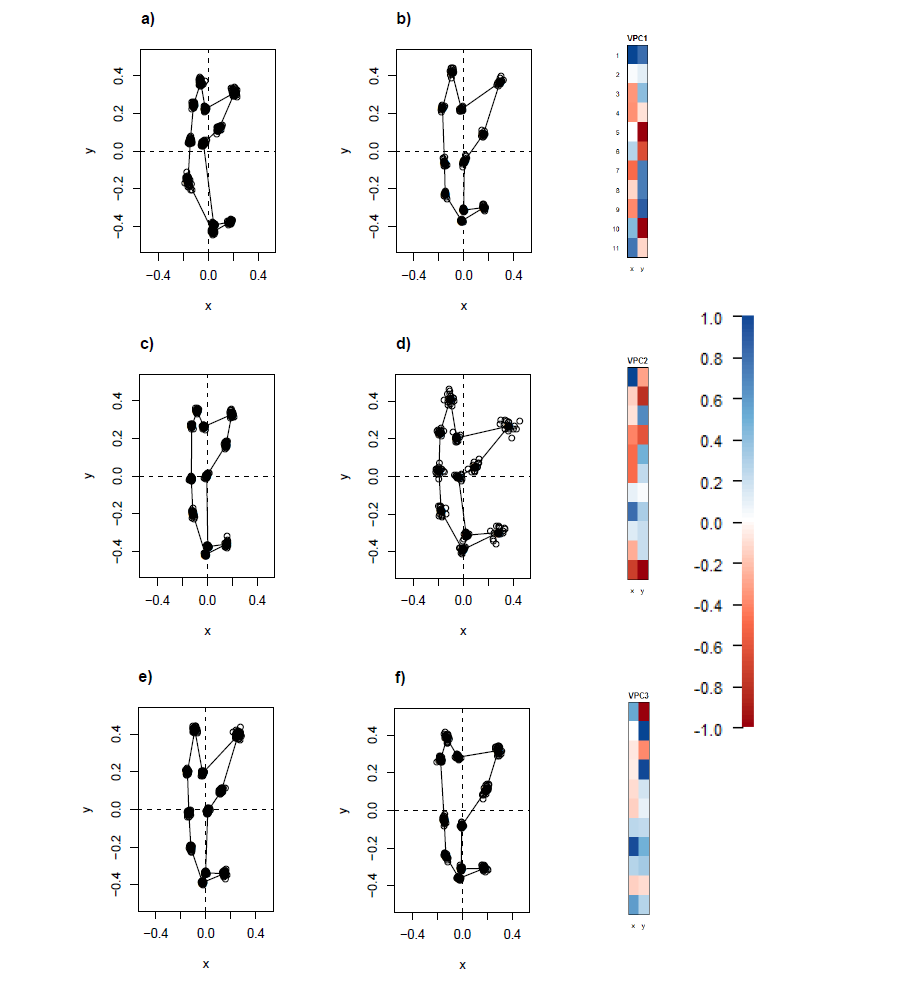

Supplement: Figure S18 — Generalized Procrustes Analysis (GPA) landmark configurations of ventral anchors of selected species with relatively large negative and positive (left and right columns, respectively) values of PC1, PC2 and PC3. The heat maps show loadings of the shape variables for the three PCs. Shape variables with important loadings: 5y, 6y, 10y (point compartment) for PC1; 1x, 1y, 2y, 3y, 4y, 11x, 11y (root compartment) for PC2; 1y, 2y, 4y (root compartment) for PC3. (A) L. chelatus; (B) L. grandis; (C) L. liewi; (D) L. bantingensis; (E) L. johorensis; (F) L. fenestrum. [file peerj-04-1668-s018.png]

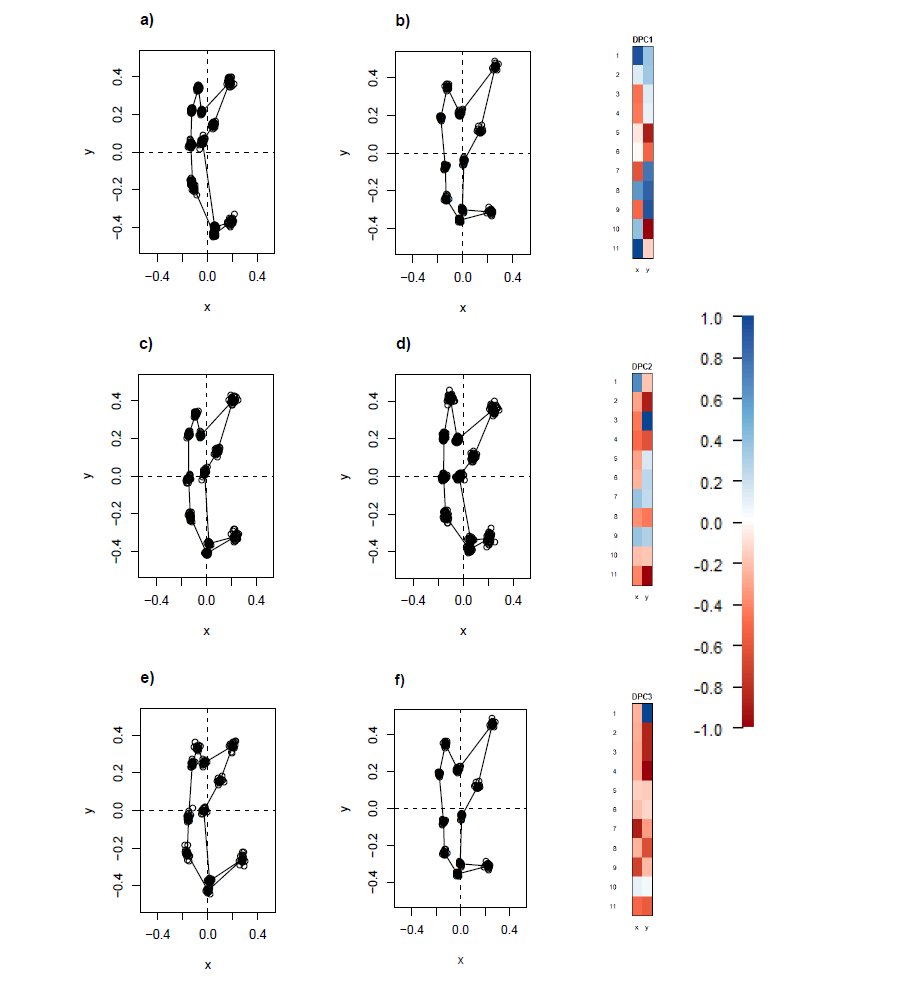

Supplement: Figure S19 — Generalized Procrustes Analysis (GPA) landmark configurations of dorsal anchors of selected species with relatively large negative and positive (left and right columns, respectively) values of PC1, PC2 and PC3. The heat maps show loadings of the shape variables for the three PCs. Shape variables with important loadings: 5y, 6y, 10y (point region) for PC1; 3y(root region) for PC2; 1y, 2y, 4y, 11y (root region) for PC3. (A) L. chelatus; (B) L. grandis; (C) L. liewi; (D) L. parvicopulatrix; (E) L. johorensis; (F) L. bantingensis. [file peerj-04-1668-s019.png]

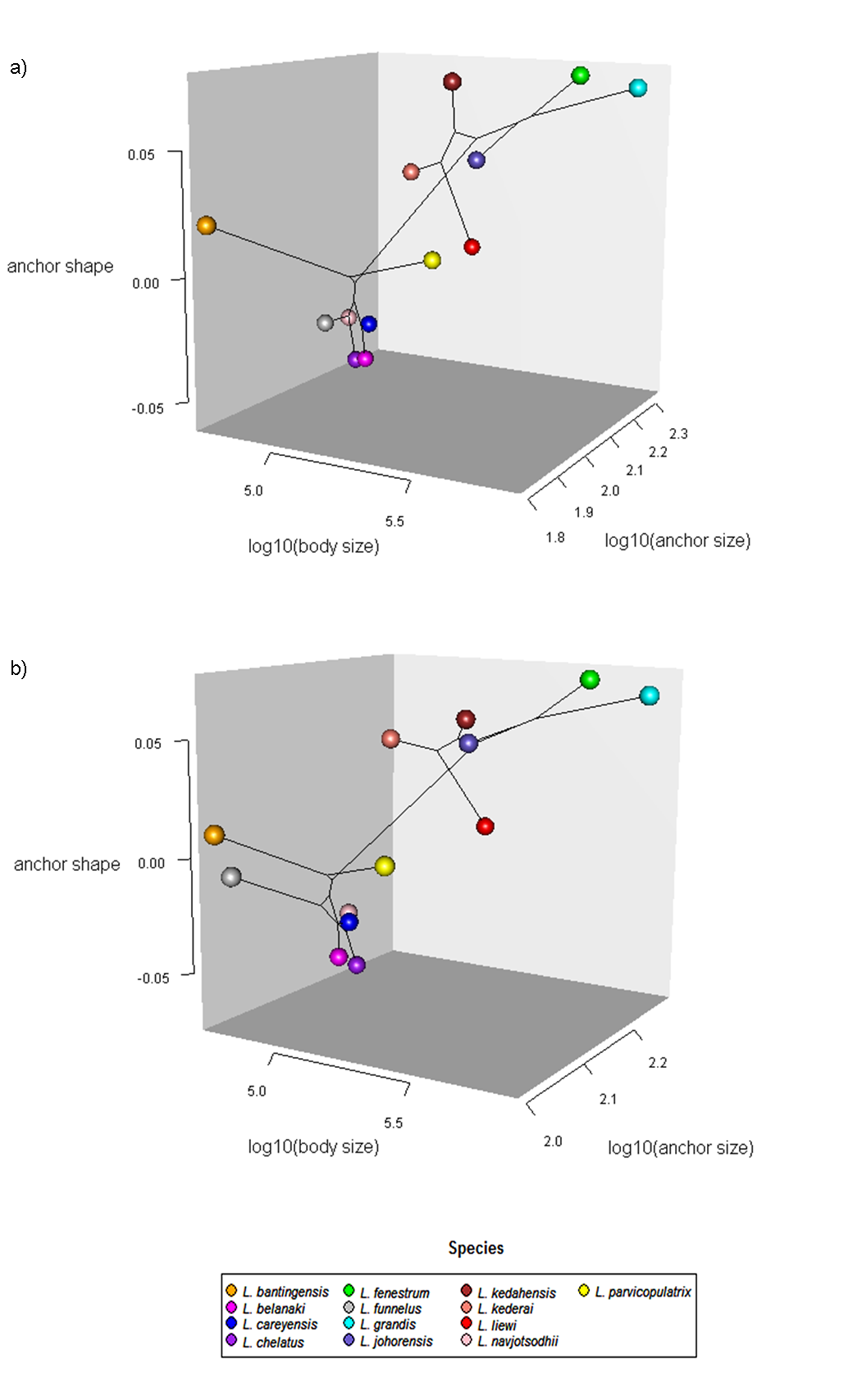

Supplement: Figure S24 — Shape (PC1 of shape variables) of (A) ventral and (B) dorsal anchors as a function of body size and anchor size (PC1 of size variables) in phylomorphospace for the 13 Ligophorus species. [file peerj-04-1668-s024.png]

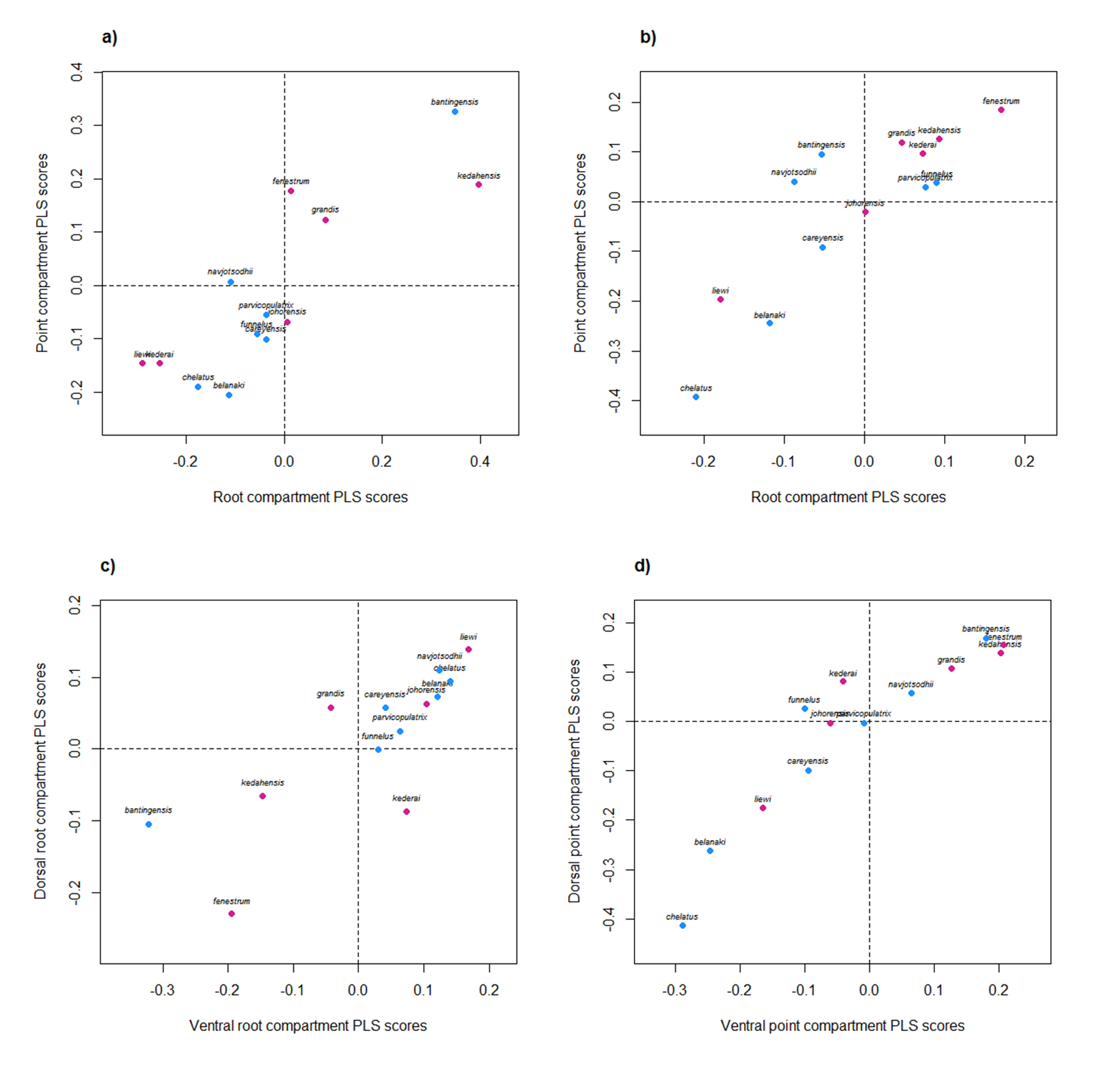

Supplement: Figure S25 — Diagnostic plots for morphological integration analysis for (A) between point and root compartments of the ventral anchors; (B) between point and root compartments of the dorsal anchors; (C) root compartments between ventral and dorsal anchors; (D) point compartments between ventral and dorsal anchors. Color legend: purple for species that infect Moolgarda buchanani; blue for species that infect Liza subviridis. [file peerj-04-1668-s025.png]

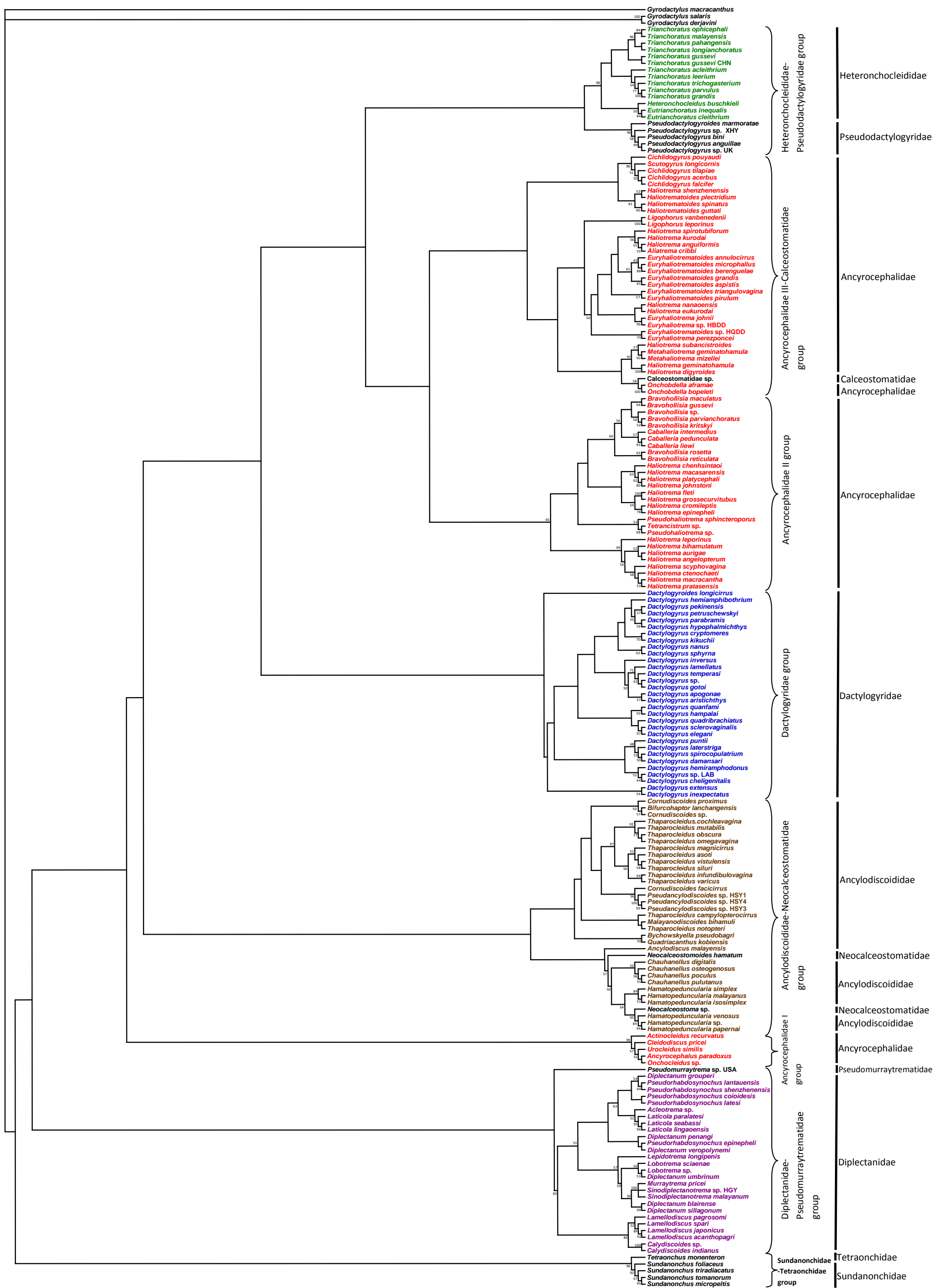

Supplement: Figure S26 — Maximum parsimony tree (PAUP, Version 4.0.b10) constructed using 28S rRNA sequences from members of different families within the order Dactylogyridea, with Gyrodactylus spp. as outgroup. Bootstrap values less than 50% are not shown. Source: Tan (2013). [file peerj-04-1668-s026.pdf]
