# Supplementary material for: Monogenean anchor morphometry: systematic value, phylogenetic signal, and evolution
Source: PeerJ. 2016 Feb 4;4:e1668. doi: 10.7717/peerj.1668 (PMC4783769; doi:10.7717/peerj.1668)
Supplement: Table S3 — Sample sizes of each of the 13 Ligophorus species before and after quality control filtering. Not all samples that passed quality control were usable for body size estimation (third column). [file peerj-04-1668-s029.pdf]

Ligophorus species	Sample size before filtering (n=530)	Sample size after filtering (n=437)	Sample size for body size estimation (n=407)
L. bantingensis	31	17	17
L. belanaki	48	43	42
L. careyensis	10	8	8
L. chelatus	49	41	36
L. fenestrum	49	25	25
L. funnelus	49	47	47
L. grandis	22	15	14
L. johorensis	50	49	47
L. kedahensis	50	38	36
L. kederai	41	35	35
L. liewi	32	31	26
L. navjotsodhii	50	45	34
L. parvicopulatrix	49	43	40
