# Supplementary material for: Monogenean anchor morphometry: systematic value, phylogenetic signal, and evolution
Source: PeerJ. 2016 Feb 4;4:e1668. doi: 10.7717/peerj.1668 (PMC4783769; doi:10.7717/peerj.1668)
Supplement: Table S4 — Matrix of morphological character states for 13 Ligophorus species. See Table 2 for descriptions of each character. Abbreviation: A for characters in Set A; B for characters in Set B. [file peerj-04-1668-s030.pdf]

Ligophorus species	Morphological characters																	
	1	2	3	4	5	6	7		8		9		10		11		12	
							A	B	A	B	A	B	A	B	A	B	A	B
L. grandis	1	0	0	1	4	1	3	1	2	1	1	0	1	1	2	1	1	0
L. fenestrum	1	1	4	1	4	1	2	1	2	1	1	0	1	1	2	1	1	1
L. johorensis	1	1	4	1	4	1	0	1	2	1	1	0	1	0	2	1	1	0
L. kedahensis	1	1	4	1	4	0	3	1	2	1	1	0	1	1	2	1	1	0
L. kederai	1	1	4	1	4	0	0	2	2	1	1	1	1	1	1	1	1	0
L. liewi	2	1	0	1	4	0	0	1	0	1	1	0	1	1	2	1	1	0
L. chelatus	0	0	1	0	2	0	0	1	0	1	0	0	0	1	1	1	0	2
L. navjotsodhii	0	0	0	1	4	0	0	1	0	1	0	0	0	1	1	1	0	2
L. funnelus	1	1	3	0	1	1	1	1	1	1	0	1	0	1	1	1	0	1
L. belanaki	1	1	3	0	0	2	1	1	0	1	1	0	0	1	1	1	0	1
L. careyensis	0	0	2	0	0	2	1	1	0	1	1	0	0	1	2	1	1	1
L. bantingensis	1	1	3	0	1	0	3	0	2	0	0	1	0	1	0	0	0	0
L. parvicopulatrix	0	1	4	1	3	1	3	1	1	1	0	1	0	1	1	1	0	2
