# Supplementary material for: Monogenean anchor morphometry: systematic value, phylogenetic signal, and evolution
Source: PeerJ. 2016 Feb 4;4:e1668. doi: 10.7717/peerj.1668 (PMC4783769; doi:10.7717/peerj.1668)
Supplement: Table S5 — Summary statistics for anchor size, anchor shape, body size and tube length of the male copulatory organ. The states of three morphological characters (see Table 2) for the latter are also included. [file peerj-04-1668-s031.pdf]

Ligophorus species	Fish host	Mean PC1 of anchor size variables (µm)		Mean PC1 or anchor shape variables x 0.01		Median of Body size (µm)		Male copulatory organ	
		Ventral	Dorsal	Ventral	Dorsal	Length	Width	*Tube length (µm)	Morphological character states
L. grandis	*Moolgarda buchanani*	170	173	6.8	7.6	2023 (1355-2165)	325 (200-379)	59 (52-63)	100
L. fenestrum		170	170	7.8	6.4	1522 (898-2441)	270 (131-500)	86 (73-95)	114
L .johorensis		164	154	4.5	5.2	1102 (718-1311)	162 (113-239)	53 (46-60)	114
L. kedahensis		136	154	6.8	5.3	994 (694-1396)	175 (76-278)	65 (57-75)	114
L. kederai		166	148	2.8	4.2	746 (569-1006)	130 (80-223)	83 (79-87)	114
L. liewi		211	191	0.5	0.8	1055 (814-1270)	125 (90-166)	81 (71-88)	210
L. chelatus	*Liza subviridis*	164	159	-6.1	-6.7	610 (351-791)	94 (54-123)	69 (60-76)	001
L. navjotsodhii		160	157	-3.9	-4.1	570 (431-792)	97 (58-143)	71 (63-96)	000
L. funnelus		136	106	-3.8	-1.3	551 (342-874)	99 (55-147)	77 (64-85)	113
L. belanaki		163	144	-5.4	-6.1	597 (420-801)	106 (64-166)	95 (83-111)	113
L. careyensis		157	145	-4.3	-4.6	599 (488-825)	115 (83-191)	94 (78-111)	002
L. bantingensis		64	101	2.1	1.9	621 (335-804)	88 (55-148)	67 (59-79)	113
L. parvicopulatrix		135	122	-0.9	-2.3	1064 (639-1322)	140 (67-224)	48 (41-66)	014
